# Risk Factors Associated with *Toxoplasma gondii* in Patients with Cardiovascular Diseases from Western Romania

**DOI:** 10.3390/microorganisms12040673

**Published:** 2024-03-28

**Authors:** Angela Dragomir, Maria Alina Lupu, Cosmin Gheorghe Maciuceanu, Liana Maria Chicea, Tudor Rares Olariu

**Affiliations:** 1 Discipline of Parasitology, Department of Infectious Disease, Victor Babes University of Medicine and Pharmacy, 300041 Timisoara, Romania; angela.dragomir@umft.ro (A.D.); cosmin.maciuceanu18@gmail.com (C.G.M.); rolariu@umft.ro (T.R.O.); 2 Center for Diagnosis and Study of Parasitic Diseases, Department of Infectious Disease, Victor Babes University of Medicine and Pharmacy, 300041 Timisoara, Romania; 3 Clinical Laboratory, Institute of Cardiovascular Diseases, 300310 Timisoara, Romania; 4 Patogen Preventia, 300124 Timisoara, Romania; 5 Department II Medical Clinic, “Victor Papilian” Faculty of Medicine, Lucian Blaga University of Sibiu, 550024 Sibiu, Romania; liana.chicea@gmail.com; 6 Internal Medicine Department, Academic Emergency Hospital, 550245 Sibiu, Romania; 7 Clinical Laboratory, Municipal Clinical Emergency Teaching Hospital, 300254 Timisoara, Romania

**Keywords:** *Toxoplasma gondii*, antibodies, seroprevalence, risk factors, epidemiology, cardiovascular patients, Romania

## Abstract

Background: Limited data are available in the existing literature regarding the seroepidemiology of *T. gondii* infection among cardiovascular patients. We aimed to comprehensively assess the prevalence of *T. gondii* infection and associated risk factors among Romanian cardiovascular patients. Methods: Serologic testing was conducted in 1205 patients with cardiovascular diseases to demonstrate the presence of *T. gondii* antibodies. An avidity test was performed in patients with detectable IgG and IgM antibodies. A structured questionnaire was designed to identify the potential risk factors associated with *T. gondii.* Results: The overall seroprevalence of *T. gondii* antibodies was 52.1%, with the highest value observed in patients diagnosed with dilated cardiomyopathy (66.66%) and the lowest in patients with myopericarditis (30.0%). The 11 patients found with detectable IgM and IgG antibodies had a high avidity test result. A patient’s area of residence, gender, educational level, owning dogs, owning any pet, and toxoplasmosis awareness were significantly associated with *T. gondii* seropositivity in multiple logistic regression analyses. Conclusions: This study provides novel and valuable insights into the seroprevalence and risk factors associated with *T. gondii* among Romanian cardiovascular patients. Our findings reiterate the importance of toxoplasmosis awareness and health education for better control and prevention of infection with *T. gondii.*

## 1. Introduction

*Toxoplasma gondii,* an obligate intracellular protozoan, is one of the most successful and well-adapted parasites due to its ability to spread within all ecosystems and across different hosts (humans and domestic and wild animals and birds) and to infect different types of cells [1,2,3,4].

Approximately 2 billion people worldwide are chronically infected with *T. gondii*, and the prevalence varies widely (between countries and within a country) due to differences in host susceptibility, hygiene, diet, habits, and climate (higher values are observed in low-altitude areas with warm and humid climates) [3,5].

**Figure 1:**
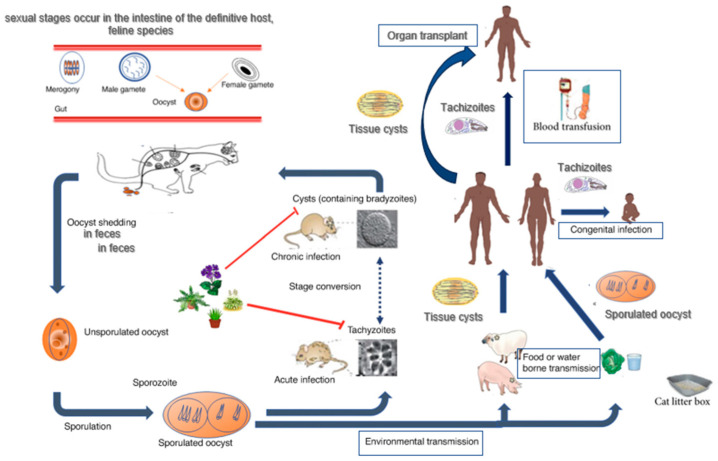
*Toxoplasma gondii*—life cycle. Adapted from Niveria et al., 2023 [10], Ahmadpour et al., 2023 [11], and Mose et al., 2020 [12].

The life cycle of *T. gondii* is complex (functioning in a prey–predator system) and involves a definitive host that harbours sexual reproduction (domestic and wild cats) and an intermediate host that harbours asexual reproduction (most warm-blooded mammals, birds, and humans) [6,7]. All three developmental stages of *T. gondii* can infect humans: (i) tachyzoites (the rapid-reproducing forms that can penetrate any nucleated cell); (ii) bradyzoites (the slow-replicating forms, found in tissue cysts), and (iii) sporozoites (found in oocysts released via felid faeces) [3,6,8,9]. Humans can become infected with *T. gondii* in four ways: (i) foodborne transmission (the consumption of raw/undercooked meat and primary offal containing bradyzoites inside tissue cysts); (ii) zoonotic transmission (the consumption of water and food contaminated with sporulated oocysts containing sporozoites); (iii) vertical transmission (tachyzoites transmitted through the placenta to the foetus); (iv) transmission via blood transfusion (in the case of a recently infected donor, parasitemic at the time of blood sampling) or organ transplant (if the organs contain tissue cysts or tachyzoites) [3,6,9] (Figure 1).

In humans, *T. gondii* exists either in the form of tachyzoites (found in acute infections) or bradyzoites (characteristic of chronic infection). Tissue cysts, found predominantly in the brain, retina, skeletal muscles, and cardiac muscles, may persist throughout life due to their ability to elude immunomediated destruction and their long-term survival [6,7].

Accurate diagnosis (through indirect and direct methods) plays a crucial role in monitoring and preventing *T. gondii* infection. In immunocompetent individuals, the presence of specific *T. gondii* antibodies (IgG, IgM, IgA) is evaluated through indirect serological methods. Anti-*T. gondii* IgG antibodies persist throughout a person’s life, and anti-*T. gondii* IgM antibodies may be detected for months or even years after infection [13]. Therefore, IgG avidity test results are highly valuable in identifying the moment of infection in pregnant women, in patients with retinochoroiditis or uveitis, and in organ donors or transplant recipients [1,7].

### Top of Form

With a loss of 2–8 million disability-adjusted life years, toxoplasmosis is one of the most damaging zoonotic diseases in the world [5]. The course of infection and its severity are influenced by different factors: (i) the inoculated dose and the stage of the parasite (bradyzoites vs. sporozoites); (ii) the parasite genotype; (iii) the genetic characteristics of the host; and (iv) the host’s immune status [1]. In immunocompetent individuals, a generally asymptomatic infection (rarely requiring intervention) may be fatal in the case of an atypical strain [2,5]. In immunocompromised patients (those with HIV/AIDS or undergoing cancer treatment or organ transplant), toxoplasmosis may be life-threatening, while in pregnant women, it may lead to miscarriage and can cause significant disease in congenitally infected newborns and infants [2,3,14].

Over time, there has been increasing interest in evaluating the potential association between *T. gondii* infection and other pathologies: (i) psychiatric disorders (major depression, schizophrenia, bipolar disorder, epilepsy, personality changes) [4,15,16,17]; (ii) neurologic diseases (Parkinson’s and Alzheimer’s diseases) [18,19]; and (iii) different types of cancer, including brain tumours [20,21,22].

The potential cardiac damage associated with *T. gondii* infection presents as atrial and ventricular arrhythmias, pericardial effusion, constrictive pericarditis, myocarditis, and acute heart failure [23]. The World Health Organization (WHO) states that cardiovascular diseases (CVDs) are the leading cause of mortality worldwide, with approximately 17.9 million deaths each year (an estimated 32% of all global deaths) [24]. The cardiovascular involvement in toxoplasmosis is often asymptomatic or overshadowed by neurological manifestations. Limited data are available in the existing international literature regarding the seroepidemiology of *T. gondii* infection among cardiovascular patients [25,26]. We have previously assessed the seroprevalence of toxoplasmosis in cardiovascular patients from Western Romania [27]. However, no evaluation regarding the potential risk factors associated with *T. gondii* in cardiovascular patients has previously been conducted. Therefore, in this study, we aimed to comprehensively assess the risk factors associated with the seroprevalence of *T. gondii* in patients with cardiovascular diseases.

## 2. Materials and Methods

### 2.1. Study Design and Population

We enrolled 1205 consecutive volunteer patients diagnosed with cardiovascular diseases in the order that they were admitted to the Institute of Cardiovascular Diseases in Timisoara, between July and October 2019. The Institute of Cardiovascular Diseases provides specialized healthcare services to the inhabitants of Western Romania (with a total population of 2,110,963 located in 5 counties: Arad, Bihor, Caras-Severin, Hunedoara, and Timis) and is considered a reference medical institution, with an average of 5000 treated patients annually. Clinical diagnoses were established based on the *International Classification of Diseases 10th Revision* (ICD-10) [28].

At study enrolment, venous blood samples were collected into Clot Activator Vacuum and Serum Separation Gel Tubes and centrifugated at 4000× *g* for 10 min, in maximum 30 min after collection. The sera were then transferred into sterile Centrifuge Eppendorf Tubes and stored at −20 °C until they were tested for IgG and/or IgM *T. gondii* antibodies. Serum samples with detectable *T. gondii* antibodies were further tested to evaluate the presence of *T. gondii* IgM antibodies only. In cases of positive serologic IgG and IgM test results, a specific IgG avidity test was performed.

The study participants’ demographic data (age, gender, area of residence) were extracted from the electronic database of the Institute of Cardiovascular Diseases, using a code (without their identification). All data have been processed with utmost confidentiality. An interviewer-administered, structured questionnaire was designed for this study to identify the potential risk factors associated with *T. gondii*: educational level (elementary/middle school, high school, university), employment status, owning cat(s), number of cats owned, cleaning cat litter, owning dog(s), number of dogs owned, owning cat(s) and/or dog(s), contact with soil (through agriculture and/or gardening activities), gardening with gloves, eating unwashed raw vegetables/fruits, the consumption of raw/undercooked meat, drinking raw/unpasteurised milk, drinking alcohol, smoking habits and drinking water. Study participants were also questioned regarding toxoplasmosis awareness and their personal medical history: previous blood transfusions and associated chronic diseases (such as diabetes, neurological diseases, gastrointestinal diseases, liver diseases, or cancer). Regarding smoking status, current and/or former smokers were recorded as a yes and those who had never smoked were recorded as a no. Study participants were grouped according to their age (at the time of enrolment in the study) in 7 age groups: 19–29 years, 30–39 years, 40–49 years, 50–59 years, 60–69 years, 70–79 years, and 80 years and over.

### 2.2. Serologic Tests

All serum samples were tested at the Center for Diagnosis and Study of Parasitic Diseases, Victor Babes University of Medicine and Pharmacy, Timisoara, Romania. A Pastorex Toxo kit (Bio-Rad, Marnes-la-Coquette, France) was used to simultaneously detect the presence of IgG and/or IgM antibodies to *T. gondii.* Previous reports showed an excellent ability to detect *T. gondii* antibodies in patients with acute and chronic toxoplasmosis for this latex particle agglutination test [29,30,31].

To identify the presence of serum anti-*T. gondii* IgM antibodies and evaluation of IgG avidity, the enzyme-linked fluorescent assay (ELFA) designed for VIDAS (bioMérieux, Marcy-l’Etoile, France) was used. ELFA IgM (VIDAS Toxo IgM kit) has a sensitivity of 100% and a specificity of 98.6% [32,33]. An accuracy of 93.4% in detecting a *T. gondii* infection dating more than 4 months was recently found for IgG avidity Vidas [34]. Quality controls and testing and interpretation of results were based on the manufacturer’s criteria.

### 2.3. Interpretation of the Serologic Test Results

*T. gondii* IgM test results were interpreted as follows: <0.55, negative; ≥0.55 to 0.65, equivocal, and >0.65, positive [35]. For the purposes of this study, equivocal test results were considered negative.

The Vidas IgG avidity test was interpreted as follows: <0.2, low avidity; ≥0.2 to 0.29, equivocal result; >0.3%, high avidity [35]. In case of low or equivocal test results, the *T. gondii* infection may have occurred within the 4 months before testing. The possibility of a primary infection within the previous 4 months was excluded by a high avidity test result [36].

### 2.4. Data Management and Statistical Analysis

All collected data were introduced in a Microsoft Excel database, version 2011 (Microsoft Corp., Redmond, WA, USA). Statistical analyses were performed using MedCalc for Windows, version 19.4 (MedCalc Software, Ostend, Belgium) and Epi Info statistical package, version 3.3.2 (Centers for Disease Control and Prevention, Atlanta, GA, USA). Odds ratios (ORs) with 95% confidence intervals (CIs) were calculated. To identify the significant association between *T. gondii* seroprevalence and risk factors and to compare proportions between groups, we used Mantel–Haenszel chi-square test and Fisher’s 2-tailed exact test. *p*-values < 0.05 were considered to be of statistical significance. Variables that reached a significance level in the univariable analyses were further analysed using the multivariable logistic regression model.

### 2.5. Ethical Consideration

The protocol of this study was conducted in accordance with the Helsinki Declaration and approved by the Victor Babes University Ethics Committee, Timisoara, Romania (no. 6 from 16 March 2018). All study participants were thoroughly informed about the study goals and procedures and provided written informed consent.

## 3. Results

The 1205 adults diagnosed with cardiovascular diseases enrolled in the study were aged between 19 and 94 years (mean age = 64.34 ± 12.06 years), 703 (58.3%) were residents of urban areas, and 779 (64.6%) were males (Table 1).

*T. gondii* IgG and/or IgM antibodies were demonstrated in 52.1% (628/1205) of study participants (95% CI: 49.29–54.93). Of the 628 study participants with detectable *T. gondii* antibodies, 11 (1.75%) were identified as having *T. gondii* IgM antibodies, and the IgG avidity test was subsequently performed in those 11 samples. A high avidity test result (≥0.3) was obtained for all samples tested. In the 617 (98.25%) of the 628 cases in which the IgG avidity test was not performed, the diagnosis of chronic infection was based on negative test results for *T. gondii* IgM antibodies.

The *T. gondii* seroprevalence showed a significant age-associated increase, from 26.32% (5/19) in the age group of 19–29 years to 54.55% (54/99) in the age group of 40–49 years (*p* = 0.04), to 53.37% (222/416) in the age group of 60–69 years (*p* = 0.03), to 54.49% (194/356) in the age group of 70–79 years (*p* = 0.01), and 59.77% (52/87) in patients aged 80 years and over (*p* = 0.01) (Table 1).

The prevalence of *T. gondii* infection was significantly higher in patients residing in rural areas (57.57%, 289/502) compared to those from urban areas (48.22%, 339/703) (*p* = 0.001) and in females (56.34%, 240/426) compared to males (49.81%, 388/779) (*p* = 0.03) (Table 1).

When data were analysed according to the level of education, the *T. gondii* seroprevalence decreased with an increase in level of education, from 58.04% in patients who graduated from elementary/middle school to 33.33% in those who attended university. Moreover, the seroprevalence was significantly higher in study participants who attended primary/middle school (58.04%, 260/448) and those who attended high school (51.41%, 329/640) compared to patients who attended university (33.33%, 39/117) (*p* < 0.001) (Table 2).

We found that retired study participants were 1.37 times more likely to test positive for *T. gondii* antibodies compared to employed ones (*p* = 0.009) (Table 2).

Owning cats, owning dogs, and owning any pets (cats and/or dogs) were variables that were significantly associated with *T. gondii* seroprevalence in our study group (*p* < 0.001, *p* = 0.01, and *p* < 0.001, respectively). However, there was no significant relationship between the number of cats owned, the cleaning of cat litter, the number of dogs owned, and seropositivity for *T. gondii* (Table 2).

Contact with soil (through agriculture or gardening activities), gardening with gloves, eating unwashed raw vegetables and/or fruits, the consumption of raw/undercooked meat, drinking raw/unpasteurized milk, drinking alcohol, smoking habits, type of water consumed were all found not to be risk factors for *T. gondii* infection in patients with cardiovascular diseases in univariate analysis (Table 2).

In our study group, only 166 (13.77%) patients had an awareness of toxoplasmosis. The *T. gondii* seroprevalence was significantly higher in study participants who had never heard or read about toxoplasmosis (53.99%, 561/1039) compared to those who had (40.36%, 67/166) (*p* = 0.001) (Table 2).

No significant differences in *T. gondii* seroprevalence were observed in univariate analysis regarding patients’ history of blood transfusions and associated chronic diseases (Table 2).

When the variables identified as risk factors for *T. gondii* infection in the univariate analysis (age, area of residence, gender, educational level, employment status, owning cats, owning dogs, owning any pets, and awareness of toxoplasmosis) were evaluated using a multiple logistic regression model, only the area of residence, gender, educational level, owning dogs, owning any pet, and toxoplasmosis awareness remained significantly associated with *T. gondii* seropositivity (Table 3).

When data were analysed according to the ICD-10 Diagnosis Code, the highest *T. gondii* seroprevalence was observed in patients diagnosed with dilated cardiomyopathy (66.66%, 20/30) and the lowest in patients with myopericarditis (30.0%, 3/10) (Table 4).

## 4. Discussion

There is little information in the international literature regarding the epidemiology of *T. gondii* infection in patients with cardiovascular diseases. Most of the currently available data comprise the case reports of immunocompromised patients (transplant recipients or individuals infected with HIV) [26]. Moreover, to the best of our knowledge, this is the first study that has evaluated the potential risk factors associated with *T. gondii* infection in Romanian cardiovascular patients.

When we compared our results with those from studies conducted in similar groups of patients with cardiovascular diseases, we found that the 52.1% *T. gondii* seroprevalence found in our study is higher than the 13.8% seroprevalence found in Mexico [25], but lower than the 63.1% and 63.73% prevalences reported in Egypt [37] and Iran [38], respectively. It is well known that the prevalence of *T. gondii* infection varies widely across the globe [4,7,39], and there are several factors that can explain this phenomenon: (i) host susceptibility; (ii) cultural habits; (iii) cooking habits and diet; (iv) hygiene; (v) the presence of cats and their number; (vi) the conditions in the external environment that may or may not favour the survival of the infecting oocysts of *T. gondii*; and (vii) socioeconomic conditions [1,4,39]. Additionally, the differences observed may be explained by variations in the sample size of the study groups and different assays (with different specificities and/or sensitivities) used to evaluate the presence of *T. gondii* antibodies [40].

The univariate analysis revealed that the prevalence of *T. gondii* antibodies increased with age in our study group, due to a prolonged length of exposure to the parasite [40]. However, when multiple logistic regression was performed, we noticed that age was no longer a significant risk factor for toxoplasmosis. In their study, Alvarado-Esquivel and colleagues also found no association between age and seropositivity for *T. gondii* [25]. The diverse backgrounds of study participants and individual circumstances may lead to different ages of exposure to the parasite and may explain the result [41].

Area of residence and gender were both found to be associated with *T. gondii* infection in our survey, suggesting that residing in a rural area and being female lead to a greater risk of becoming infected. Activities carried out in rural areas (gardening, farming, handling animals) may expose individuals to the main sources of infection with *T. gondii*: (i) oocysts excreted with cat faeces and (ii) tissue cysts found in meat. Feral cats defecate in sandboxes or gardens, posing a risk of infection for some individuals, regardless of whether they own a cat [42,43]. The finding that *T. gondii* seroprevalence is significantly higher in females compared to males in Romanian cardiovascular patients represents important information for public health. Women spend more time cooking and therefore handle raw meat more frequently [44]. Additionally, they typically take care of, and tend to play more with, animals (including cats) [45].

Educational level was found to be an important risk factor for the occurrence of *T. gondii* infection in our study group: the multiple logistic regression analysis revealed that a lower educational level significantly increases the seropositivity for *T. gondii*. In patients with heart diseases from Durango City, Mexico, a low educational level was not associated with the prevalence of *T. gondii* infection [25]. However, the present study validates our previous findings [40,44] suggesting that a high level of education (university) increases the awareness and understanding of *T. gondii* infection and its preventive measures, therefore reducing the likelihood of exposure [40].

In Romanian patients with cardiovascular diseases, retired individuals were more likely to present anti-*T. gondii* antibodies compared to employed ones when using univariate analysis. However, when multiple logistic regression was performed, employment status was no longer identified as a risk factor for *T. gondii*, and this is in agreement with previous findings [25].

Similar to another report [25], in our study group, owning cats was found to be associated with *T. gondii* seropositivity when using univariate analysis. However, after performing the multiple logistic regression, contact with cats was no longer identified as a risk factor for *T. gondii*, and this validates our previous findings in Romanian blood donors [40]. Interestingly, owning dogs and owning any pet (cats and/or dogs) may increase the risk for *T. gondii* infection in our study group. Previous reports revealed that 25% of dogs from Central Romania (Cluj-Napoca) [46] and 63% from Southern Romania are infected with *T. gondii* [47]. Dogs may act as mechanical carriers for *T. gondii* oocysts and could play an important role in the transmission of this parasite (i) through it contaminating their fur and (ii) through feeding on cat faces (accidentally ingesting *T. gondii* oocysts and defecating them, probably after passive gastrointestinal transport) [48,49,50]. The identification of owning pets (cats and/or dogs) being a risk factor for *T. gondii* infection in patients with cardiovascular diseases confirms our previous result found in Romanian pregnant women [44]. The *T. gondii* seroprevalence in household cats from Central and North-Western Romania was 47% [51]. The close contact between cats and dogs (the most popular pet animals worldwide) and their owner may explain the potential risk factor for *T. gondii* infection in humans represented by these animals [52].

In this study, *T. gondii* seroprevalence was not found to be associated with the consumption of raw or undercooked meat; this is similar to recently data published by other authors [25]. Moreover, this result confirms our previous findings in Romanian blood donors and pregnant women [40,44]. This outcome could potentially be explained by several factors: (i) the increase in the utilization of frozen and industrially processed meat and meat products; (ii) the implementation of modern systems and enhanced hygiene conditions in animal farms; (iii) improved sanitary measures during meat processing [40,53].

Contact with soil, eating unwashed raw vegetables/fruits, drinking raw/unpasteurized milk, and the type of water used for drinking were not found to be risk factors for *T. gondii* infection in Romanian patients with cardiovascular diseases. Previous reports found no association between seropositivity for *T. gondii* and the consumption of untreated water [25,38] or of unwashed raw vegetables/fruits [25] in patients with heart diseases. Khademvatan and colleagues found that exposure to soil and drinking raw milk were significantly associated with *T. gondii* infection in their study [38].

In contrast with findings observed by Alvarado-Esquivel and colleagues [25], we found no association between *T. gondii* seropositivity and the consumption of alcohol. Further studies are needed to elucidate the potential role of alcohol consumption in *T. gondii* infection.

Our results revealed that the vast majority of study participants (86.22%) were not aware of *T. gondii* infection. Moreover, toxoplasmosis awareness was found to be associated with *T. gondii* seropositivity in multiple logistic regression analysis. The present study underlines the importance of formal education and literacy in increasing the awareness of *T. gondii* infection and methods for its prevention.

Previous reports have documented the possibility of *T. gondii* transmission via the transfusion of leukocytes or platelets and blood transfusion from asymptomatic seropositive individuals in the early stages of acute infection (*T. gondii* can survive for more than 50 days in citrated blood at 5 °C) [40,54]. However, we noticed that the prevalence of *T. gondii* was not associated with a history of blood transfusion in our study group; this is similar to the results previously published by Alvarado-Esquivel and colleagues [25].

The present study has several limitations. Despite the large size of the study sample, the number of patients with detectable *T. gondii* IgM antibodies was small, and, consequently, the number of serum samples tested for avidity was limited. Serum samples with negative results for *T. gondii* IgG antibodies were not tested further to assess the presence of *T. gondii* IgM antibodies. However, it is exceptionally uncommon to detect IgM antibodies in the absence of IgG antibodies [55]. Therefore, it is highly unlikely that the results of this study would have significantly changed had negative IgG sera been tested for IgM. Female patients are less represented in our study, and this can be listed as another limitation. Gender differences are increasingly recognized as influencing susceptibility and pathology in cardiovascular diseases due to several factors: sex chromosomes, gonadal hormones, different cardiac structure and function, intrinsic variations in cardiac and vascular aging, differences in myocardial substrate metabolism, and cultural and social behaviours [56,57]. Properly evaluating *T. gondii*’s seroprevalence and the influence of the parasite on cardiac function was difficult in patients diagnosed with dilated cardiomyopathy, aortic aneurysm, hypertension, congestive heart failure, pulmonary oedema, pulmonary embolism, and myopericarditis due to the limited number of study participants. Further studies involving a larger participant pool will be needed to validate the results of the present survey.

## 5. Conclusions

This study provides novel and valuable insights into the seroprevalence and risk factors associated with *T. gondii* among Romanian patients with cardiovascular diseases. Area of residence, gender, educational level, owning dogs, owning any pet, and toxoplasmosis awareness were found to be significantly associated with *T. gondii* seropositivity in a multiple logistic regression analysis. Our findings reiterate the importance of toxoplasmosis awareness and health education for better control and prevention of infection with *T. gondii.*

## Figures and Tables

**Table 1:** Seroprevalence of *Toxoplasma gondii* infection in patients with cardiovascular diseases from Western Romania according to age, area of residence, and gender.

		Prevalence of *T. gondii* Infection Univariate Analysis		
Variables	No. Tested	N (%)	OR (95% CI)	*p*
**Age group (years)**				
19–29	19	5 (26.32%)	1 (Ref.)	
30–39	25	12 (48.00%)	2.58 (0.71–9.36)	0.21
40–49	99	54 (54.55%)	3.36 (1.12–10.04)	**0.04**
50–59	203	89 (43.84%)	2.18 (0.75–6.29)	0.15
60–69	416	222 (53.37%)	3.20 (1.13–9.05)	**0.03**
70–79	356	194 (54.49%)	3.35 (1.18–9.50)	**0.01**
≥80	87	52 (59.77%)	4.16 (1.37–12.59)	**0.01**
**Area of residence**				
Urban	703	339 (48.22%)	1 (Ref.)	
Rural	502	289 (57.57%)	1.45 (1.15–1.83)	**0.001**
**Gender**				
Male	779	388 (49.81%)	1 (Ref.)	
Female	426	240 (56.34%)	1.30 (1.02–1.64)	**0.03**
**Total**	1205	628 (52.1%)	-	-

N, number of *T. gondii* seropositive study participants; OR, odds ratio; CI, confidence interval; Ref., reference.

**Table 2:** Factors associated with *Toxoplasma gondii* seroprevalence in patients with cardiovascular diseases from Western Romania.

		Prevalence of *T. gondii* Antibodies Univariate Analysis		
Variables	No. Tested	N (%)	OR (95% CI)	*p*
**Educational level**				
Primary/middle school	448	260 (58.04%)	2.76 (1.80–4.24)	**<0.001**
High school	640	329 (51.41%)	2.11 (1.39–3.20)	**<0.001**
University	117	39 (33.33%)	1 (Ref.)	-
**Employment status**				
Employed	429	201 (46.85%)	1 (Ref.)	-
Unemployed	10	7 (70.00%)	2.64 (0.67–10.37)	0.20
Retired	766	420 (54.83%)	1.37 (1.08–1.74)	**0.009**
**Owning cat(s)**				
No	554	259 (46.75%)	1 (Ref.)	-
Yes	651	369 (56.68%)	1.49 (1.18–1.87)	**<0.001**
**Number of cats owned**				
1	596	335 (56.21%)	1 (Ref.)	-
2	23	14 (60.87%)	1.21 (0.51–2.84)	0.83
3	13	7 (53.85%)	0.90 (0.30–2.73)	1.0
≥4	19	13 (68.42%)	1.68 (0.63–4.50)	0.35
**Cleaning cat litter**				
No	314	180 (57.32%)	1 (Ref.)	-
Yes	337	189 (56.08%)	0.95 (0.69–1.29)	0.75
**Owning dog(s)**				
No	722	356 (49.31%)	1 (Ref.)	-
Yes	483	272 (56.31%)	1.32 (1.05–1.67)	**0.01**
**Number of dogs owned**				
1	241	138 (57.26%)	1 (Ref.)	-
2	215	121 (56.28%)	0.96 (0.66–1.39)	0.85
3	21	9 (42.86%)	0.55 (0.22–1.37)	0.25
≥4	6	4 (66.67%)	1.49 (0.26–8.30)	1.0
**Owning cat(s) and/or dog(s)**				
No	442	194 (43.89%)	1 (Ref.)	-
Yes	763	434 (56.88%)	1.68 (1.33–2.13)	**<0.001**
**Contact with soil**				
No	732	378 (51.64%)	1 (Ref.)	-
Yes	473	250 (52.85%)	1.04 (0.83–1.32)	0.72
**Gardening with gloves**				
No	414	216 (52.17%)	1 (Ref.)	-
Yes	59	34 (57.63%)	1.24 (0.71–2.16)	0.48
**Eating unwashed raw vegetables/fruits**				
No	550	286 (52.00%)	1 (Ref.)	-
Yes	655	342 (52.21%)	1.00 (0.80–1.26)	0.95
**Consumption of raw/undercooked meat**				
No	785	396 (50.45%)	1 (Ref.)	-
Yes	420	232 (55.24%)	1.21 (0.95–1.53)	0.11
**Drinking raw/unpasteurized milk**				
No	1060	545 (51.42%)	1 (Ref.)	-
Yes	145	83 (57.24%)	1.26 (0.89–1.79)	0.21
**Drinking alcohol**				
No	551	282 (51.18%)	1 (Ref.)	-
Yes	654	346 (52.91%)	1.07 (0.85–1.34)	0.56
**Smoking habit**				
No	956	501 (52.41%)	1 (Ref.)	-
Yes	249	127 (51.00%)	0.94 (0.71–1.24)	0.72
**Source of drinking** **water**				
Bottled water	21	9 (42.86%)	1 (Ref.)	-
Private drilling	411	226 (54.99%)	1.62 (0.67–3.95)	0.36
Public water supply	773	393 (50.84%)	1.37 (0.57–3.31)	0.51
**Toxoplasmosis awareness**				
No	1039	561 (53.99%)	1.73 (1.24–2.41)	**0.001**
Yes	166	67 (40.36%)	1 (Ref.)	-
**History of blood transfusions**				
No	1144	599 (52.36%)	1 (Ref.)	-
Yes	61	29 (47.54%)	0.82 (0.49–1.38)	0.51
**Associated chronic diseases**				
No	1098	564 (51.37%)	1 (Ref.)	-
Yes	107	64 (59.81%)	1.40 (0.94–2.11)	0.10

N, number of *T. gondii* seropositive study participants; OR, odds ratio; CI, confidence interval; Ref., reference.

**Table 3:** Risk factors for *Toxoplasma gondii* infection in patients with cardiovascular diseases from Western Romania (multiple logistic regression analysis).

Variables	OR (95% CI)	*p*
**Area of residence**		
Urban	1 (Ref.)	-
Rural	1.33 (1.04–1.7)	**0.02**
**Gender**		
Male	1 (Ref.)	-
Female	1.30 (1.01–1.66)	**0.03**
**Educational level**		
Primary/middle school	3.04 (1.83–5.03)	**<0.001**
High school	2.89 (1.83–4.54)	**<0.001**
University	1 (Ref.)	-
**Owning dog(s)**		
No	1 (Ref.)	-
Yes	0.60 (0.40–0.90)	**0.01**
**Owning cat(s) and/or dog(s)**		
No	1 (Ref.)	-
Yes	2.80 (1.59–4.93)	**<0.001**
**Toxoplasmosis awareness**		
No	1 (Ref.)	
Yes	0.62 (0.43–0.87)	**0.007**

OR, odds ratio; CI, confidence interval; Ref., reference.

**Table 4:** Seroprevalence of *Toxoplasma gondii* antibodies in patients with cardiovascular diseases from Western Romania, according to their ICD-10 Diagnosis Code (univariate analysis).

Diagnosis	ICD-10 Diagnosis Code	No. Tested	Prevalence of *T. gondii* Antibodies Univariate Analysis		
			No. Tested Positive (%)	OR (95% CI)	*p*
Dilated cardiomyopathy	I42.0	30	20 (66.66%)	4.66 (0.98–22.01)	0.06
Atherosclerotic heart disease	I25.1	175	97 (55.42%)	2.90 (0.72–11.59)	0.19
Aortic aneurysm	I71	13	7 (53.84%)	2.72 (0.47–15.46)	0.40
Arrhythmia	I49.9	171	92 (53.80%)	2.71 (0.67–10.86)	0.19
Valvular heart disease	I05–I09	132	70 (53.03%)	2.63 (0.65–10.63)	0.19
Hypertension	I10	68	35 (51.47%)	2.47 (0.59–10.37)	0.31
Congestive heart failure	I50	86	44 (51.16%)	2.44 (0.59–10.08)	0.31
Unstable angina	I20.0	323	165 (51.08%)	2.43 (0.61–9.58)	0.21
Acute myocardial infarction	I21	192	93 (48.43%)	2.19 (0.55–8.72)	0.33
Pulmonary oedema or pulmonary embolism	J81.1–I26	5	2 (40.00%)	1.55 (0.16–14.65)	1
Myopericarditis	I30–I40	10	3 (30.00%)	1 (Ref.)	-

OR, odds ratio; CI, confidence interval; Ref., reference.

## Data Availability

Data are contained within the article.
